# 1-[(6-Chloro­pyridin-3-yl)meth­yl]-10-nitro-1,2,3,5,6,7,8,9-octa­hydro-5,9-methano­imidazo[1,2-*a*]azocin-5-ol

**DOI:** 10.1107/S1600536813014402

**Published:** 2013-06-08

**Authors:** Shu-Xia Cui, Guang-You Zhang, Zhong-Zhen Tian

**Affiliations:** a Shandong Provincial Key Laboratory of Fluorine Chemistry and Chemical Materials, School of Chemistry and Chemical Engineering, University of Jinan, People’s Republic of China

## Abstract

In the title compound, C_16_H_19_ClN_4_O_3_, the cyclo­hexane ring displays a chair formation and the tetra­hydro­pyridine ring displays an envelope conformation with the methyl­ene C atom as the flap; the imidazolidine ring also displays an envelope conformation with a methyl­ene C atom as the flap. In the crystal, O—H⋯N hydrogen bonds between hy­droxy groups and pyridine rings link inversion-related mol­ecules into dimers. Weak C—H⋯O hydrogen bonds further link the dimers into supra­molecular chains running along the *c* axis.

## Related literature

For background to the title compound, see: Jeschkel & Nauen (2008 ▶). For the synthesis, see: Tian *et al.* (2007 ▶).
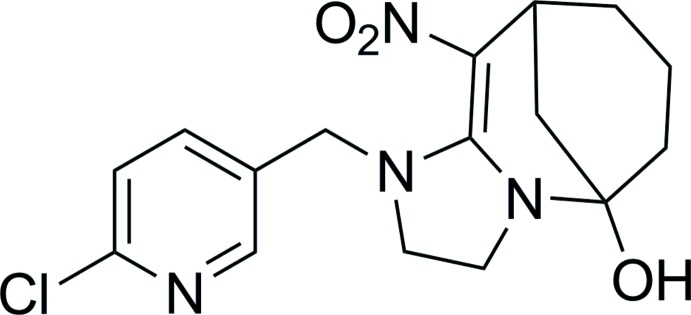


## Experimental

### 

#### Crystal data

C_16_H_19_ClN_4_O_3_
*M* *_r_* = 350.80
Monoclinic, 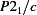

*a* = 13.3975 (14) Å
*b* = 18.7124 (18) Å
*c* = 6.5721 (8) Å
β = 97.897 (10)°
*V* = 1632.0 (3) Å^3^
*Z* = 4
Mo *K*α radiation
μ = 0.26 mm^−1^
*T* = 296 K
0.38 × 0.24 × 0.23 mm

#### Data collection

Bruker APEXII diffractometer
Absorption correction: multi-scan (*SADABS*; Bruker, 2001 ▶) *T*_min_ = 0.93, *T*_max_ = 0.94
7705 measured reflections
2921 independent reflections
1727 reflections with *I* > 2σ(*I*)
*R*_int_ = 0.056

#### Refinement

*R*[*F* ^2^ > 2σ(*F* ^2^)] = 0.071
*wR*(*F* ^2^) = 0.217
*S* = 1.05
2921 reflections
217 parameters
H-atom parameters constrained
Δρ_max_ = 0.33 e Å^−3^
Δρ_min_ = −0.34 e Å^−3^

### 

Data collection: *APEX2* (Bruker, 2007 ▶); cell refinement: *SAINT* (Bruker, 2007 ▶); data reduction: *SAINT*; program(s) used to solve structure: *SHELXTL* (Sheldrick, 2008 ▶); program(s) used to refine structure: *SHELXTL*; molecular graphics: *SHELXTL*; software used to prepare material for publication: *SHELXTL*.

## Supplementary Material

Crystal structure: contains datablock(s) I, global. DOI: 10.1107/S1600536813014402/xu5695sup1.cif

Structure factors: contains datablock(s) I. DOI: 10.1107/S1600536813014402/xu5695Isup2.hkl

Additional supplementary materials:  crystallographic information; 3D view; checkCIF report

## Figures and Tables

**Table 1:** Hydrogen-bond geometry (Å, °)

*D*—H⋯*A*	*D*—H	H⋯*A*	*D*⋯*A*	*D*—H⋯*A*
O3—H3⋯N4^i^	0.82	2.04	2.855 (4)	174
C11—H11*A*⋯O1^ii^	0.97	2.53	3.467 (5)	161
C13—H13⋯O2^ii^	0.93	2.48	3.265 (5)	142

Symmetry codes: (i) 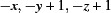; (ii) 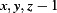.

**Fig. 1.**
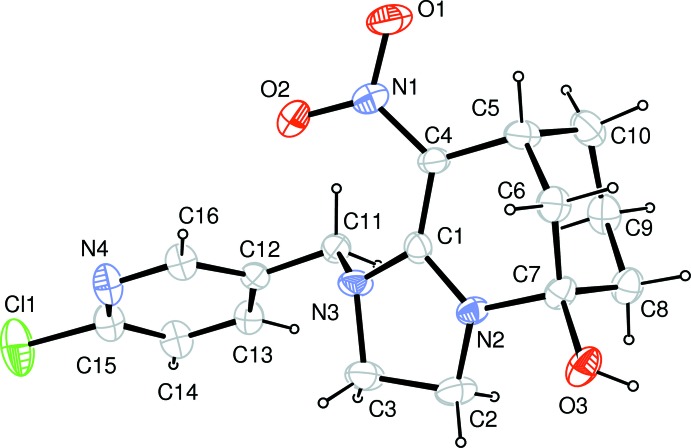
The molecular structure of the title compound with atom numbering scheme. Displacement ellipsoids are drawn at the 30% probability level. The H atoms are shown as circles of arbitrary size.

## supplementary crystallographic information

### Comment

The past decades have witnessed the great power and versatile ability of neonicotinoids as a novel class of insecticides (Jeschkel & Nauen, 2008), Several commercial neonicotinoid compounds were launched successively in the latest two decades, and they presented a broad spectrum performance in controlling various insects. Our interest was introducing bicyclic struture to fix the direction of the nitro group, and synthesized a class of novel *cis*-neonicotinoid compounds with carbon bicyclic, in which the title compound exhibited good insecticidal activities against pea aphids.

The structure of compound is shown in Fig. 1 with the atom-numbering scheme. In the title compound, the the cyclohexane ring displays a chair formation while the hexahydroazocine ring displays an envelope conformation with the methylene C atom on the flap; the imidazolidine ring also displays an envelope confromation with a methylene C atom on the flap. In the crystal, intermolecular O—H···N hydrogen bonds between hydroxyl groups and pyridine rings link inversion-related molecules into dimer. Weak C—H···O hydrogen bonds further link the dimers into the supramolecular arichiecture.

### Experimental

The title compound was synthesized according to the literature (Tian *et al.*, 2007). Single crystals suitable for X-ray analysis were obtained by slow evaporation of the solution of dichloromethane and ether of the title compound.

### Refinement

Hydroxyl H atom was located in a difference map and refined with distance restraints of O—H = 0.82 Å, *U*_iso_(H) = 1.5*U*_eq_(O). Other H atoms were positioned geometrically and refined using a riding model with C—H = 0.93–0.98 Å, *U*_iso_(H) = 1.2*U*_eq_(C).

### Crystal data

**Table:**

C_16_H_19_ClN_4_O_3_	*F*(000) = 736
*M**_r_* = 350.80	*D*_x_ = 1.428 Mg m^−^^3^
Monoclinic, *P*2_1_/*c*	Mo *K*α radiation, λ = 0.71073 Å
Hall symbol: -P 2ybc	Cell parameters from 1166 reflections
*a* = 13.3975 (14) Å	θ = 2.6–25.2°
*b* = 18.7124 (18) Å	µ = 0.26 mm^−^^1^
*c* = 6.5721 (8) Å	*T* = 296 K
β = 97.897 (10)°	Prism, colourless
*V* = 1632.0 (3) Å^3^	0.38 × 0.24 × 0.23 mm
*Z* = 4	

### Data collection

**Table:**

Bruker APEXII diffractometer	2921 independent reflections
Radiation source: fine-focus sealed tube	1727 reflections with *I* > 2σ(*I*)
Graphite monochromator	*R*_int_ = 0.056
Detector resolution: 16.03 pixels mm^-1^	θ_max_ = 25.2°, θ_min_ = 2.7°
φ and ω scans	*h* = −16→16
Absorption correction: multi-scan (*SADABS*; Bruker, 2001)	*k* = −21→22
*T*_min_ = 0.93, *T*_max_ = 0.94	*l* = −7→6
7705 measured reflections	

### Refinement

**Table:**

Refinement on *F*^2^	Primary atom site location: structure-invariant direct methods
Least-squares matrix: full	Secondary atom site location: difference Fourier map
*R*[*F*^2^ > 2σ(*F*^2^)] = 0.071	Hydrogen site location: inferred from neighbouring sites
*wR*(*F*^2^) = 0.217	H-atom parameters constrained
*S* = 1.05	*w* = 1/[σ^2^(*F*_o_^2^) + (0.1042*P*)^2^ + 0.1363*P*] where *P* = (*F*_o_^2^ + 2*F*_c_^2^)/3
2921 reflections	(Δ/σ)_max_ = 0.001
217 parameters	Δρ_max_ = 0.33 e Å^−^^3^
0 restraints	Δρ_min_ = −0.34 e Å^−^^3^

### Special details

**Table:**

Geometry. All esds (except the esd in the dihedral angle between two l.s. planes) are estimated using the full covariance matrix. The cell esds are taken into account individually in the estimation of esds in distances, angles and torsion angles; correlations between esds in cell parameters are only used when they are defined by crystal symmetry. An approximate (isotropic) treatment of cell esds is used for estimating esds involving l.s. planes.
Refinement. Refinement of F^2^ against ALL reflections. The weighted R-factor wR and goodness of fit S are based on F^2^, conventional R-factors R are based on F, with F set to zero for negative F^2^. The threshold expression of F^2^ > 2sigma(F^2^) is used only for calculating R-factors(gt) etc. and is not relevant to the choice of reflections for refinement. R-factors based on F^2^ are statistically about twice as large as those based on F, and R-factors based on ALL data will be even larger.

### Fractional atomic coordinates and isotropic or equivalent isotropic displacement parameters (Å^2^)

**Table:**

	*x*	*y*	*z*	*U*_iso_*/*U*_eq_	
Cl1	−0.41200 (9)	0.65584 (9)	0.0781 (3)	0.0997 (6)	
N1	0.1452 (3)	0.65779 (16)	0.7861 (5)	0.0462 (9)	
N2	0.2189 (2)	0.53441 (15)	0.4048 (5)	0.0366 (8)	
N3	0.0721 (2)	0.59219 (15)	0.3726 (5)	0.0437 (8)	
N4	−0.2457 (2)	0.63479 (18)	0.3227 (6)	0.0571 (10)	
O1	0.1870 (2)	0.69232 (16)	0.9378 (5)	0.0673 (9)	
O2	0.0505 (2)	0.65481 (15)	0.7522 (5)	0.0588 (8)	
O3	0.35157 (19)	0.45270 (13)	0.4224 (4)	0.0510 (8)	
H3	0.3216	0.4252	0.4901	0.077*	
C1	0.1637 (3)	0.58526 (18)	0.4877 (6)	0.0373 (9)	
C2	0.1721 (3)	0.5161 (2)	0.1983 (6)	0.0533 (11)	
H2A	0.1769	0.4653	0.1716	0.064*	
H2B	0.2017	0.5427	0.0949	0.064*	
C3	0.0639 (3)	0.5387 (2)	0.2069 (7)	0.0563 (12)	
H3A	0.0341	0.5594	0.0774	0.068*	
H3B	0.0233	0.4984	0.2386	0.068*	
C4	0.2050 (3)	0.62191 (19)	0.6634 (6)	0.0388 (9)	
C5	0.3162 (3)	0.61790 (18)	0.7288 (6)	0.0443 (10)	
H5	0.3302	0.6297	0.8751	0.053*	
C6	0.3494 (3)	0.54049 (19)	0.6983 (6)	0.0455 (10)	
H6A	0.4208	0.5352	0.7467	0.055*	
H6B	0.3123	0.5080	0.7752	0.055*	
C7	0.3281 (3)	0.52359 (18)	0.4689 (6)	0.0388 (9)	
C8	0.3893 (3)	0.57185 (19)	0.3484 (7)	0.0477 (10)	
H8A	0.3741	0.5609	0.2032	0.057*	
H8B	0.4605	0.5630	0.3904	0.057*	
C9	0.3664 (3)	0.65028 (19)	0.3831 (7)	0.0515 (11)	
H9A	0.4128	0.6797	0.3188	0.062*	
H9B	0.2987	0.6609	0.3177	0.062*	
C10	0.3751 (3)	0.6691 (2)	0.6090 (7)	0.0564 (12)	
H10A	0.3502	0.7173	0.6229	0.068*	
H10B	0.4455	0.6682	0.6680	0.068*	
C11	0.0317 (3)	0.66343 (18)	0.3105 (6)	0.0400 (9)	
H11A	0.0647	0.6811	0.1980	0.048*	
H11B	0.0465	0.6963	0.4248	0.048*	
C12	−0.0792 (3)	0.66095 (16)	0.2453 (6)	0.0347 (9)	
C13	−0.1215 (3)	0.68244 (19)	0.0504 (6)	0.0450 (10)	
H13	−0.0801	0.6977	−0.0435	0.054*	
C14	−0.2246 (3)	0.6813 (2)	−0.0049 (7)	0.0539 (11)	
H14	−0.2539	0.6961	−0.1346	0.065*	
C15	−0.2822 (3)	0.6576 (2)	0.1372 (8)	0.0518 (11)	
C16	−0.1457 (3)	0.6367 (2)	0.3742 (7)	0.0495 (11)	
H16	−0.1190	0.6208	0.5044	0.059*	

### Atomic displacement parameters (Å^2^)

**Table:**

	*U*^11^	*U*^22^	*U*^33^	*U*^12^	*U*^13^	*U*^23^
Cl1	0.0386 (7)	0.1346 (13)	0.1228 (14)	0.0014 (7)	0.0000 (7)	0.0343 (10)
N1	0.055 (2)	0.042 (2)	0.042 (2)	0.0066 (15)	0.0090 (17)	−0.0048 (16)
N2	0.0401 (17)	0.0310 (17)	0.0390 (18)	0.0055 (12)	0.0071 (14)	−0.0050 (14)
N3	0.0452 (19)	0.0319 (17)	0.052 (2)	0.0061 (13)	−0.0022 (15)	−0.0140 (16)
N4	0.039 (2)	0.056 (2)	0.078 (3)	−0.0026 (15)	0.0113 (18)	0.014 (2)
O1	0.081 (2)	0.070 (2)	0.048 (2)	0.0147 (15)	0.0004 (16)	−0.0235 (16)
O2	0.0499 (19)	0.071 (2)	0.059 (2)	0.0059 (14)	0.0197 (14)	−0.0108 (15)
O3	0.0569 (18)	0.0363 (16)	0.064 (2)	0.0123 (12)	0.0222 (14)	0.0068 (13)
C1	0.034 (2)	0.032 (2)	0.047 (2)	−0.0027 (15)	0.0071 (16)	−0.0003 (18)
C2	0.067 (3)	0.042 (2)	0.049 (3)	0.0120 (19)	0.002 (2)	−0.012 (2)
C3	0.057 (3)	0.046 (2)	0.062 (3)	0.0052 (18)	−0.007 (2)	−0.017 (2)
C4	0.042 (2)	0.041 (2)	0.033 (2)	0.0023 (16)	0.0033 (16)	−0.0090 (17)
C5	0.050 (2)	0.041 (2)	0.040 (2)	0.0053 (17)	−0.0041 (18)	−0.0069 (18)
C6	0.044 (2)	0.045 (2)	0.046 (3)	0.0069 (17)	0.0016 (18)	0.0036 (19)
C7	0.045 (2)	0.026 (2)	0.047 (2)	0.0073 (15)	0.0114 (18)	0.0043 (17)
C8	0.044 (2)	0.046 (2)	0.055 (3)	0.0071 (17)	0.0116 (19)	0.005 (2)
C9	0.050 (2)	0.037 (2)	0.069 (3)	0.0002 (17)	0.012 (2)	0.014 (2)
C10	0.043 (2)	0.043 (2)	0.080 (3)	−0.0032 (17)	−0.003 (2)	−0.007 (2)
C11	0.043 (2)	0.033 (2)	0.046 (2)	−0.0023 (15)	0.0127 (17)	−0.0021 (17)
C12	0.041 (2)	0.0219 (18)	0.041 (2)	−0.0019 (14)	0.0067 (17)	0.0027 (16)
C13	0.045 (2)	0.040 (2)	0.050 (3)	0.0013 (16)	0.0093 (18)	0.0104 (19)
C14	0.050 (3)	0.057 (3)	0.053 (3)	0.002 (2)	0.001 (2)	0.018 (2)
C15	0.043 (2)	0.043 (2)	0.069 (3)	0.0056 (17)	0.007 (2)	0.013 (2)
C16	0.046 (2)	0.047 (2)	0.056 (3)	−0.0001 (17)	0.005 (2)	0.008 (2)

### Geometric parameters (Å, º)

**Table:**

Cl1—C15	1.729 (4)	C5—H5	0.9800
N1—O1	1.254 (4)	C6—C7	1.529 (5)
N1—O2	1.258 (4)	C6—H6A	0.9700
N1—C4	1.386 (5)	C6—H6B	0.9700
N2—C1	1.363 (5)	C7—C8	1.515 (5)
N2—C2	1.456 (5)	C8—C9	1.523 (5)
N2—C7	1.480 (4)	C8—H8A	0.9700
N3—C1	1.357 (4)	C8—H8B	0.9700
N3—C3	1.472 (5)	C9—C10	1.515 (6)
N3—C11	1.475 (4)	C9—H9A	0.9700
N4—C15	1.320 (6)	C9—H9B	0.9700
N4—C16	1.337 (5)	C10—H10A	0.9700
O3—C7	1.407 (4)	C10—H10B	0.9700
O3—H3	0.8200	C11—C12	1.490 (5)
C1—C4	1.391 (5)	C11—H11A	0.9700
C2—C3	1.518 (5)	C11—H11B	0.9700
C2—H2A	0.9700	C12—C16	1.387 (5)
C2—H2B	0.9700	C12—C13	1.387 (5)
C3—H3A	0.9700	C13—C14	1.380 (5)
C3—H3B	0.9700	C13—H13	0.9300
C4—C5	1.495 (5)	C14—C15	1.364 (6)
C5—C10	1.527 (6)	C14—H14	0.9300
C5—C6	1.537 (5)	C16—H16	0.9300
			
O1—N1—O2	119.6 (3)	N2—C7—C8	110.8 (3)
O1—N1—C4	118.7 (3)	O3—C7—C6	113.1 (3)
O2—N1—C4	121.6 (3)	N2—C7—C6	107.2 (3)
C1—N2—C2	110.4 (3)	C8—C7—C6	110.5 (3)
C1—N2—C7	123.4 (3)	C7—C8—C9	111.2 (3)
C2—N2—C7	121.0 (3)	C7—C8—H8A	109.4
C1—N3—C3	108.5 (3)	C9—C8—H8A	109.4
C1—N3—C11	120.6 (3)	C7—C8—H8B	109.4
C3—N3—C11	115.0 (3)	C9—C8—H8B	109.4
C15—N4—C16	117.1 (4)	H8A—C8—H8B	108.0
C7—O3—H3	109.5	C10—C9—C8	112.4 (3)
N3—C1—N2	110.0 (3)	C10—C9—H9A	109.1
N3—C1—C4	129.9 (3)	C8—C9—H9A	109.1
N2—C1—C4	120.0 (3)	C10—C9—H9B	109.1
N2—C2—C3	101.0 (3)	C8—C9—H9B	109.1
N2—C2—H2A	111.6	H9A—C9—H9B	107.9
C3—C2—H2A	111.6	C9—C10—C5	112.6 (3)
N2—C2—H2B	111.6	C9—C10—H10A	109.1
C3—C2—H2B	111.6	C5—C10—H10A	109.1
H2A—C2—H2B	109.4	C9—C10—H10B	109.1
N3—C3—C2	104.0 (3)	C5—C10—H10B	109.1
N3—C3—H3A	110.9	H10A—C10—H10B	107.8
C2—C3—H3A	110.9	N3—C11—C12	111.4 (3)
N3—C3—H3B	110.9	N3—C11—H11A	109.4
C2—C3—H3B	110.9	C12—C11—H11A	109.4
H3A—C3—H3B	109.0	N3—C11—H11B	109.4
N1—C4—C1	121.8 (3)	C12—C11—H11B	109.4
N1—C4—C5	119.4 (3)	H11A—C11—H11B	108.0
C1—C4—C5	118.7 (3)	C16—C12—C13	116.4 (3)
C4—C5—C10	112.7 (3)	C16—C12—C11	122.2 (3)
C4—C5—C6	107.8 (3)	C13—C12—C11	121.4 (3)
C10—C5—C6	110.2 (4)	C14—C13—C12	120.4 (4)
C4—C5—H5	108.7	C14—C13—H13	119.8
C10—C5—H5	108.7	C12—C13—H13	119.8
C6—C5—H5	108.7	C15—C14—C13	117.7 (4)
C7—C6—C5	107.8 (3)	C15—C14—H14	121.2
C7—C6—H6A	110.2	C13—C14—H14	121.2
C5—C6—H6A	110.2	N4—C15—C14	124.4 (4)
C7—C6—H6B	110.2	N4—C15—Cl1	115.8 (3)
C5—C6—H6B	110.2	C14—C15—Cl1	119.8 (3)
H6A—C6—H6B	108.5	N4—C16—C12	124.0 (4)
O3—C7—N2	108.0 (3)	N4—C16—H16	118.0
O3—C7—C8	107.2 (3)	C12—C16—H16	118.0
			
C3—N3—C1—N2	3.5 (4)	C1—N2—C7—C8	−87.0 (4)
C11—N3—C1—N2	−132.3 (3)	C2—N2—C7—C8	65.2 (4)
C3—N3—C1—C4	−177.8 (4)	C1—N2—C7—C6	33.7 (4)
C11—N3—C1—C4	46.5 (6)	C2—N2—C7—C6	−174.1 (3)
C2—N2—C1—N3	13.0 (4)	C5—C6—C7—O3	−177.5 (3)
C7—N2—C1—N3	167.8 (3)	C5—C6—C7—N2	−58.5 (4)
C2—N2—C1—C4	−165.9 (3)	C5—C6—C7—C8	62.4 (4)
C7—N2—C1—C4	−11.1 (5)	O3—C7—C8—C9	178.1 (3)
C1—N2—C2—C3	−22.7 (4)	N2—C7—C8—C9	60.5 (4)
C7—N2—C2—C3	−178.2 (3)	C6—C7—C8—C9	−58.3 (4)
C1—N3—C3—C2	−17.4 (4)	C7—C8—C9—C10	51.5 (4)
C11—N3—C3—C2	121.1 (3)	C8—C9—C10—C5	−50.4 (5)
N2—C2—C3—N3	23.3 (4)	C4—C5—C10—C9	−65.2 (4)
O1—N1—C4—C1	−177.1 (3)	C6—C5—C10—C9	55.2 (4)
O2—N1—C4—C1	5.0 (5)	C1—N3—C11—C12	−162.0 (3)
O1—N1—C4—C5	7.4 (5)	C3—N3—C11—C12	64.9 (4)
O2—N1—C4—C5	−170.6 (3)	N3—C11—C12—C16	58.0 (4)
N3—C1—C4—N1	20.4 (6)	N3—C11—C12—C13	−121.5 (4)
N2—C1—C4—N1	−160.9 (3)	C16—C12—C13—C14	2.0 (5)
N3—C1—C4—C5	−164.0 (4)	C11—C12—C13—C14	−178.5 (3)
N2—C1—C4—C5	14.7 (5)	C12—C13—C14—C15	−0.8 (6)
N1—C4—C5—C10	−103.5 (4)	C16—N4—C15—C14	1.5 (6)
C1—C4—C5—C10	80.8 (4)	C16—N4—C15—Cl1	−178.9 (3)
N1—C4—C5—C6	134.7 (4)	C13—C14—C15—N4	−1.1 (7)
C1—C4—C5—C6	−41.0 (5)	C13—C14—C15—Cl1	179.3 (3)
C4—C5—C6—C7	63.1 (4)	C15—N4—C16—C12	0.0 (6)
C10—C5—C6—C7	−60.2 (4)	C13—C12—C16—N4	−1.7 (5)
C1—N2—C7—O3	155.9 (3)	C11—C12—C16—N4	178.8 (3)
C2—N2—C7—O3	−51.9 (4)		

### Hydrogen-bond geometry (Å, º)

**Table:**

*D*—H···*A*	*D*—H	H···*A*	*D*···*A*	*D*—H···*A*
O3—H3···N4^i^	0.82	2.04	2.855 (4)	174
C11—H11*A*···O1^ii^	0.97	2.53	3.467 (5)	161
C13—H13···O2^ii^	0.93	2.48	3.265 (5)	142

Symmetry codes: (i) −*x*, −*y*+1, −*z*+1; (ii) *x*, *y*, *z*−1.
